# *E. coli* infection disrupts the epithelial barrier and activates intrinsic neurosecretory reflexes in the pig colon

**DOI:** 10.3389/fphys.2023.1170822

**Published:** 2023-06-02

**Authors:** Sara Traserra, Sergi Casabella-Ramón, Patri Vergara, Marcel Jimenez

**Affiliations:** ^1^ Department of Cell Biology, Physiology and Immunology, Universitat Autònoma de Barcelona, Barcelona, Spain; ^2^ Centro de Investigación Biomédica en Red de Enfermedades Hepaticas y Digestivas (CIBERehd), Instituto de Salud Carlos III, Madrid, Spain

**Keywords:** ETEC, neurosecretion, ENS, colon, pig

## Abstract

This study aims to assess the barrier integrity and possible activation of enteric neural pathways associated with secretion and motility in the pig colon induced by an enterotoxigenic *Escherichia coli* (ETEC) challenge. 50 Danbred male piglets were used for this study. 16 were challenged with an oral dose of the ETEC strain F4+ 1.5 × 10^9^ colony-forming unit. Colonic samples were studied 4- and 9-days post-challenge using both a muscle bath and Ussing chamber. Colonic mast cells were stained with methylene blue. In control animals, electrical field stimulation induced neurosecretory responses that were abolished by tetrodotoxin (10^−6^M) and reduced by the combination of atropine (10^−4^M) and α-chymotrypsin (10U/mL). Exogenous addition of carbachol, vasoactive intestinal peptide, forskolin, 5-HT, nicotine, and histamine produced epithelial Cl^−^ secretion. At day 4 post-challenge, ETEC increased the colonic permeability. The basal electrogenic ion transport remained increased until day 9 post-challenge and was decreased by tetrodotoxin (10^−6^M), atropine (10^−4^M), hexamethonium (10^−5^M), and ondansetron (10^−5^M). In the muscle, electrical field stimulation produced frequency-dependent contractile responses that were abolished with tetrodotoxin (10^−6^M) and atropine (10^−6^M). Electrical field stimulation and carbachol responses were not altered in ETEC animals in comparison with control animals at day 9 post-challenge. An increase in mast cells, stained with methylene blue, was observed in the mucosa and submucosa but not in the muscle layer of ETEC-infected animals on day 9 post-challenge. ETEC increased the response of intrinsic secretory reflexes and produced an impairment of the colonic barrier that was restored on day 9 post-challenge but did not modify neuromuscular function.

## 1 Introduction

The enteric nervous system (ENS) is organized in two major ganglionated plexuses, the myenteric plexus (MP) and the submucosal plexus (SMP), which interconnect and regulate both motor and secretory functions (Moeser and Blikslager, 2007; Greenwood-Van Meerveld and Johnson, 2017). Enteric neurons located within the intestinal wall respond to various pathological factors, including inflammatory processes, bacterial infections, and toxins (Vasina et al., 2006). The anatomical organization of the ENS appears to be more complex in larger animals than in small laboratory animals. In the porcine colon, the submucosal plexus is divided into an inner submucosal plexus (ISP) on the epithelial side and an outer submucosal plexus (OSP) that is adjacent to the internal side of the circular muscle (Petto et al., 2015; Mazzoni et al., 2021). For this reason, and because of the physiological similarity between the porcine and the human gastrointestinal (GI) system, the pig has been considered a suitable model to study neural mechanisms related to GI functions (Hens et al., 2000; Brown and Timmermans, 2004; Gonzalez et al., 2015).

*Ex vivo* techniques are used to assess intestinal mechanisms that can be affected by bacterial toxins, such as an increase in mucosal ion transport, a leaky intestinal barrier, or impaired contractility. Furthermore, electrical field stimulation (EFS) stimulates enteric neurons, thus evaluating the contribution of the ENS to gut responses. In muscular preparations, selective pharmacological conditions and stimulation parameters must be applied to obtain an inhibitory or excitatory response (Mañé et al., 2015). EFS of intestinal preparations containing submucosal neurons are used to study nerve-mediated changes in mucosal ion transport (Keast et al., 1985). However, submucosal neural-mediated responses have been mainly evaluated in small laboratory animals with a single-layered submucosal plexus (Timmermans et al., 2001).

Nowadays, ETEC infection is still a major cause of morbidity and mortality worldwide, especially in low-income countries. Once ETEC colonization is established, the bacteria produces toxins that can directly act on enterocytes but also interfere with the ENS, thus resulting in moderate-to-severe watery diarrhea, both in humans and animals (Dubreuil, 2012; Thiagarajah et al., 2015; Zhu et al., 2017). In the pig production system, ETEC-induced diarrhea results in reduced performance, increased mortality, and therefore, in a reduction of productivity, especially in the postweaning period (Fairbrother et al., 2005; Moeser et al., 2007a; Kim et al., 2022).

Therefore, the aims of the present study were to assess the neural mechanisms associated with GI secretory and motor functions in the pig colon and to evaluate functional disruptions induced by an ETEC challenge in the post-weaning period. Since there were differences between results obtained in laboratory animals and in humans, the first experiments were aimed at characterizing the effect of agonists and antagonists on secretory and motor function in the pig colon. A second set of experiments aimed to characterize the neurosecretory response using the antagonists tested in the first experiments. Once the neurosecretory and motor mechanisms were characterized, the experimental infection was performed, experimental groups were compared, and the previously tested antagonists were used to assess the neurosecretory response.

## 2 Materials and methods

### 2.1 Animals

A total of 50 Danbred male piglets of non-vaccinated against *E. coli* mothers (weaned at 21 d of age; BW 5.0 ± 0.35 Kg), and apparently healthy and free from disease, were acquired from a commercial farm; 18 animals were used to characterize colonic neuromuscular and neurosecretory mechanisms and 32 animals were used to study the impairment of the colonic functionality after an ETEC challenge. After weaning, animals were allowed an adaptation period of at least 7 days in the farm facility of the Universitat Autònoma de Barcelona. Piglets were group-housed in a different room for each experimental group under conventional controlled conditions with 13-h:11-h light/dark cycle and a temperature of 28°C ± 2°C. To maintain an optimal temperature, partial floor heating with the addition of a heat lamp was provided in the pen. Commercial feed and drinking water were provided *ad libitum*. Euthanasia was performed under intramuscular sedation with Xylazine (2.2 mg kg^-1^ BW; Rompun, Bayer) and Zolazepam—Tiletamine (8 mg kg^-1^ BW; Zoletil 100, Virbac) and carried out by means of intravenous sodium pentobarbital (200 mg kg^-1^ BW; Dolethal, Vetoquinol S.A.). For functional studies, samples from the distal colon were collected and transported to the laboratory in ice-cold Krebs solution previously bubbled with carbogen (95%O_2_/5%CO_2_). For histological studies, tissue was fixed in Methacarn (ethanol 60 mL, chloroform 330 mL, glacial acetic acid 10 mL). The experimental protocol was reviewed and authorized by the Ethical Committee of the Universitat Autònoma de Barcelona. ETEC experimental procedures were approved under the following code, no. CEEAH: 4026.

### 2.2 *E. coli* infection

After 7 days adaptation period, 16 piglets were challenged with a single oral dose of 6 mL of the ETEC strain F4+ (1.5 × 10^9^ colony-forming unit) following the protocol described by López-Colom et al. (2019). Briefly, the oral inoculum of the ETEC strain F4+ (positive for virulence factors F4ab, F4ac, LT, STb, and negative for EAST1 and F6, F18, F41, STa, VT1, VT2, and EAE) provided by the Diseases Laboratory of UAB (ref. 30/14–3) was prepared by overnight incubation at 37°C with shaking at 250 rpm in brain heart infusion medium (BHI, Laboratorios Conda S.A.). To confirm inoculum concentrations, serial dilutions were cultured in Luria agar and incubated overnight at 37°C. The final inoculum obtained was 2.5 × 10^8^ CFU mL^-1^. Functional studies were performed on days 4 and 9 post-challenge (N = 4 and N = 12, respectively), which represent an acute and partial recovery stage (López-Colom et al., 2019). Age- and time-matched piglets were used as controls (N = 4 on day 4 and N = 12 on day 9 post-infection). During this time, animals were regularly monitored for clinical signs and body weight changes. The normal course of the infection was confirmed by a minor decrease of body weight and a mild diarrhea after ETEC infection compared with controls.

### 2.3 Solutions and drugs

The composition of the Krebs solution was as follows (in mM): 10.10 glucose, 115.48 NaCl, 21.90 NaHCO_3_, 4.61 KCl, 1.14 NaH_2_PO_4_, 2.50 CaCl_2_ and 1.16 MgSO_4_ bubbled with a mixture of 5% CO_2_:95% O_2_ (pH 7.4). A chloride free Krebs solution was also used in secretion experiments (in mM) with the following composition: 11.10 glucose, 115.49 C_6_H_11_NaO_7_, 21.90 NaHC0_3_, 4.61 C_6_H_11_KO_7_, 1.14 NaH_2_PO_4_ x 2H_2_0, 5.02 C_12_H_22_CaO_14_*H_2_0, 1.16 MgSO_4_ x 7H_2_0 bubbled with carbogen (a mixture of 95% O2/5% CO2; pH 7.4). The following drugs were used: Nω-nitro- L-arginine (L-NNA), atropine sulphate, 2-(4-lmidazolyl)ethylamine dihydrochloride (Histamine dihydrochloride), N-[2-[[[5-[(Dimethylamino)methyl]-2-furanyl]methyl]thio]ethyl]-N′-methyl-2-nitro-1,1-ethanediamine hydrochloride (Ranitidine hydrochloride), Nicotine, α-chymotrypsin, 3-(2-Aminoethyl)-5-hydroxyindole, 5-HT, 5-hydroxytryptamine (serotonin), Hexamethonium bromide (Sigma Chemicals, St. Louis, MO, United States), (1R,2S,4S,5S)-4-[2-Iodo-6-(methylamino)-9H-purin-9-yl]-2 (phosphonooxy) byciclo [3.1.0] hexane-1-methanol dihydrogen phosphate ester tetraammonium salt (MRS2500), (2-Hydroxyethyl)trimethylammonium chloride carbamate (Cch), VIP, Tetodrotoxin (TTX), Forskolin and 1,2,3,9-Tetrahydro-9-methyl-3-[(2-methyl-1H-imidazol-1-yl)methyl]-4H-carbazol-4-one hydrochloride (Ondansetron hydrochloride) (Tocris, Bristol, United Kingdom). Stock solutions were made by dissolving drugs in distilled water except for Forskolin, which was dissolved in ethanol (96%) and L-NNA, which required sonication to be dissolved in Krebs solution.

### 2.4 Measurement of electrophysiological parameters

Preparations with colonic mucosa and submucosa were obtained by removing both muscle layers. Flat segments of 1.5 cm^2^ were mounted in Ussing chambers (World Precision Instruments, Aston, United Kingdom) and were allowed to stabilize for 30–40 min before baseline values of potential difference (PD), short circuit current (I_sc_) and conductance (G) were recorded. Strips were bilaterally bathed with 5 mL of bubbled (95% O_2_/5% CO_2_) and warmed (37°C ± 1°C) Krebs buffer. The chambers, with an exposed window surface area of 0.67 cm^2^, contained two voltage sensitive electrodes (EKV; World Precision Instruments) to monitor the PD across the tissue and two Ag-AgCl current passing electrodes (EKC; World Precision Instruments) to inject the required I_sc_ to maintain a PD of zero. A voltage step of 1 mV was applied every 30 min and the change in I_sc_ was used to calculate tissue G and its reciprocal, TEER, by Ohm’s law. Data was registered via an automated voltage/current clamp (DVC-1000; World Precision Instruments) and digitized with an analog-to-digital converter (MP150; Biopac Systems, Goleta, United States). Measurements were recorded and analyzed with Acqknowledge computer software (version 3.8.1; Biopac Systems). I_sc_ and G data were normalized to the mucosal surface area. In order to study neurosecretory mechanisms, EFS was applied through two electrodes placed on both sides of the tissue and attached to the output of a stimulator (Grass S48, Grass Instruments, Quincy, MA). EFS had a total duration of 30 s (pulse duration of 0.5 ms, 5 mA and frequencies of 0.5, 1, 5, 10 and 20 Hz). The area of the response, representing the total charge elicited by neural stimulation, was measured after EFS. Δ I_sc_ was measured after drug addition.

### 2.5 Assessment of epithelial permeability

Paracellular permeability was assessed on day 4 (N = 4) and day 9 (N = 4) post-challenge. Mucosal to basolateral flux of fluorescein isothiocyanate (FITC)-labeled dextran (FD) with a molecular weight of 4kDa (FD4; TdB Consultancy AB, Uppsala, Sweden) was measured over 60 min experimental time. After tissue stabilization, FD4 was added to the mucosal reservoir to a final concentration of 2.5 × 10^−4^ mol L^-1^. Basolateral samples (250 μL, replaced by 250 µL of Krebs buffer) were taken at 30 min intervals. The concentration of fluorescein in the samples was determined by fluorometry (Infinite F200; Tecan, Crailsheim, Germany) with an excitation wavelength of 485 nm (20 nm bandwidth) and an emission wavelength of 535 (25 nm bandwidth), against a standard curve. Readings are expressed as a percentage (%) of the total amount of FD4 added to the mucosal reservoir and the slope of the FD4 permeability curve was calculated for each experimental group.

### 2.6 Mechanical experiments

Muscle strips devoid of mucosa were used for mechanical experiments. Colonic circularly oriented muscle strips were used to characterize neuromuscular mechanisms, whereas circularly and longitudinally oriented strips were used to study the effect of the ETEC challenge. In both cases, strips were mounted in a 10 mL organ bath filled with Krebs solution at 37°C ± 1°C. A tension of 1 g was applied, and tissue was allowed to equilibrate for 1 h until spontaneous phasic contractions (SPCs) were recorded. An isometric force transducer (Harvard VF-1) connected to an amplifier was used to record the mechanical activity. Data were digitalized (25 Hz) using DATAWIN1 software (Panlab, Barcelona, Spain) coupled to an ISC-16 analog-to-digital card installed in a PC. EFS was applied through two platinum electrodes placed on the support holding the tissue and connected to the output of an electronic stimulator (Grass S88, Grass Instruments, Quincy, MA).

The area under the curve (AUC) (g min^-1^) of contractions from the baseline was measured to estimate mechanical activity before and after drug addition. In order to normalize mechanical data, responses to drugs were expressed as a percentage of the basal AUC using the following formula: 1—(AUC after drug incubation/AUC previous to drug addition), 0% being a complete cessation of spontaneous motility and 100% a mechanical recording with the same AUC as the basal activity.

To study the effect of the ETEC challenge on excitatory neuromuscular transmission, Non-nitrergic and Non-purinergic (NNNP) conditions (L-NNA 1mM and MRS2500 1 µM) were used in circularly and longitudinally oriented colonic strips and EFS was applied at 30 V (train 300 ms, pulse duration 0.4 ms) and at increasing frequencies (10, 20, 30, 40, and 50Hz). The amplitude of the EFS response was measured. In another subset of experiments, Cch dose-response was studied and compared between experimental groups. In this case, the response was analyzed by measuring the AUC of contractions and expressed as g min^-1^.

### 2.7 Mast cells staining and counting

Colonic slides were stained with polychromatic methylene blue (Rieger et al., 2013). Mucosal, submucosal and muscular and serosal mast cells (MCs) were counted in 10 random fields per subject following a blinded fashion evaluation. Counts were performed on day-9 post-ETEC challenge, N = 7 and control animals, N = 8.

### 2.8 Data analysis and statistics

4 to 8 colonic strips per animal were used to characterize colonic neurosecretory and neuromuscular mechanisms. A different experimental protocol was used for each of the strips of the same animal and data are expressed as n values that represent the total number of strips analyzed from different animals (study with agonists and neurosecretion). For the ETEC challenge study, a range from 2 to 4 strips per animal was used and data are expressed as the total number of animals used in each experimental group (N). One-way ANOVA followed by Bonferroni’s *post hoc* test was used to analyze basal electrophysiological parameters, FD4 permeability slope and the secretory and muscular effect of carbachol (Cch), vasoactive intestinal peptide (VIP), forskolin, nicotine and serotonin (5-HT). Comparison of fits after Nonlinear regression curve was used to analyze histamine effect. FD4 permeability over time, neurosecretory EFS-induced response, motor responses to excitatory EFS and Cch were compared using a Two-way ANOVA followed by Bonferroni’s multiple comparisons test. Intestinal MCs assessment and the effect of neural blockade on I_sc_ were compared through a *t*-test. In all cases, results were considered statistically significant when *p* < 0.05.

## 3 Results

### 3.1 Effect of agonists and antagonists on secretory and motor function

#### 3.1.1 Role of muscarinic receptor on secretion and motility

**FIGURE 1:**
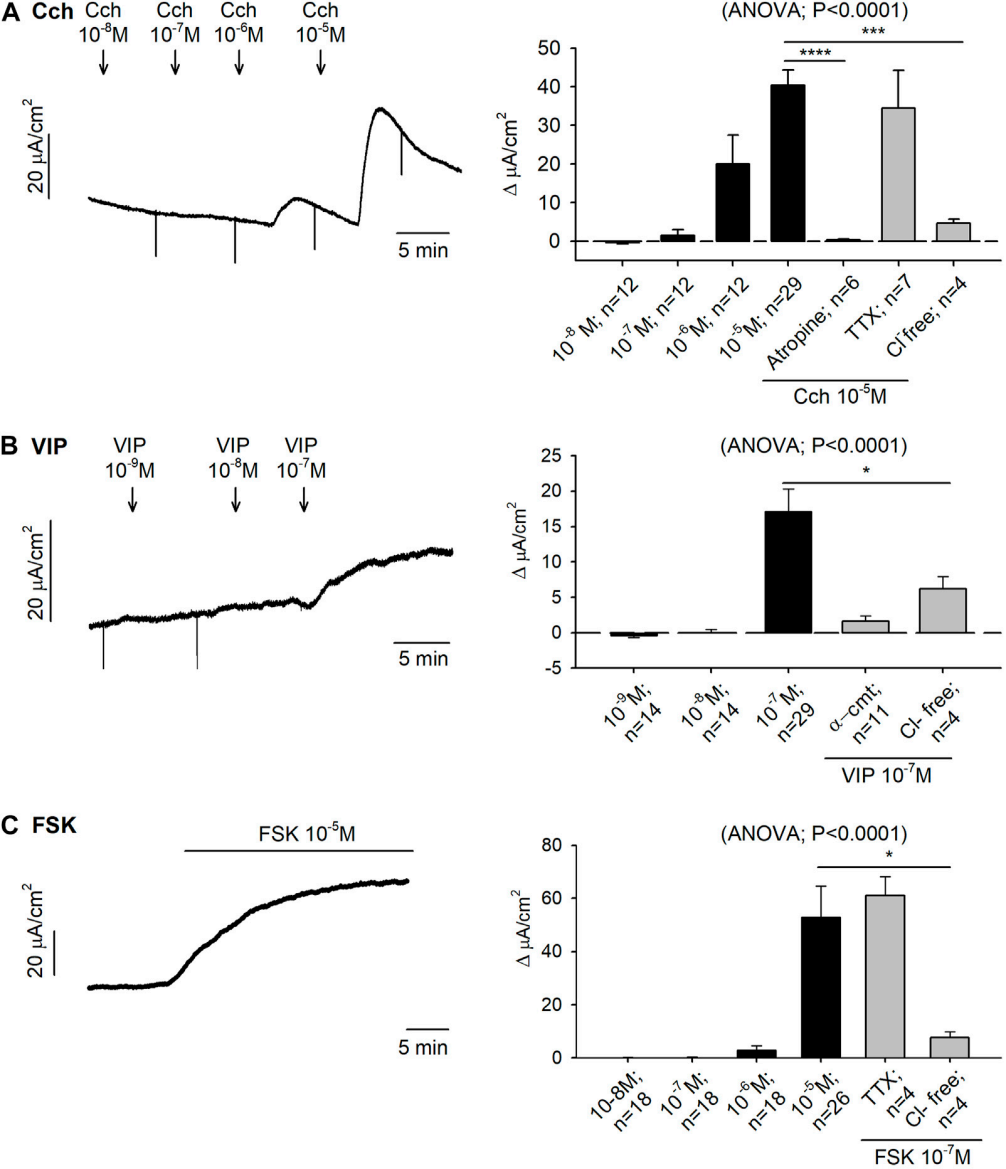
Effect of carbachol (Cch), vasoactive intestinal peptide (VIP) and forskolin (FSK) on colonic secretion. Representative current recordings (left) of accumulative concentrations of Cch **(A)**, VIP **(B)** and FSK **(C)**, and histograms showing the effect of Cch **(A)**, VIP **(B)** and FSK **(C)** in control conditions (black) and after the incubation of atropine 10^−4^ M, TTX 10^−6^ M, Cl^−^ free solution or α-cmt 10U/mL before the addition of the highest concentration tested for each drug (grey) (right). Post Hoc test after ANOVA. *, *p* < 0.05; ***, *p* < 0.001; ****, *p* < 0.0001 compared to the highest concentration tested for each drug under control conditions. Data are expressed as mean ± SEM. n values represent strips from different animals.

The cholinergic pathway was assessed in both Ussing chambers and muscle bath preparations. Serosal addition of Cch (10^–8^—10^–5^ M) caused an immediate and concentration-dependent increase in I_sc_ with an EC_50_ of 19.86 µM and reaching 40.37 ± 3.95 μA/cm^2^ ∆I_sc_ at the highest concentration tested. Pre-treatment with tetrodotoxin (TTX) 10^−6^M did not modify the Cch response, whereas atropine 10^–4^ M and Cl^−^ free Krebs solution totally blocked the Cch 10^–5^ M induced response (Figure 1A).

**FIGURE 2:**
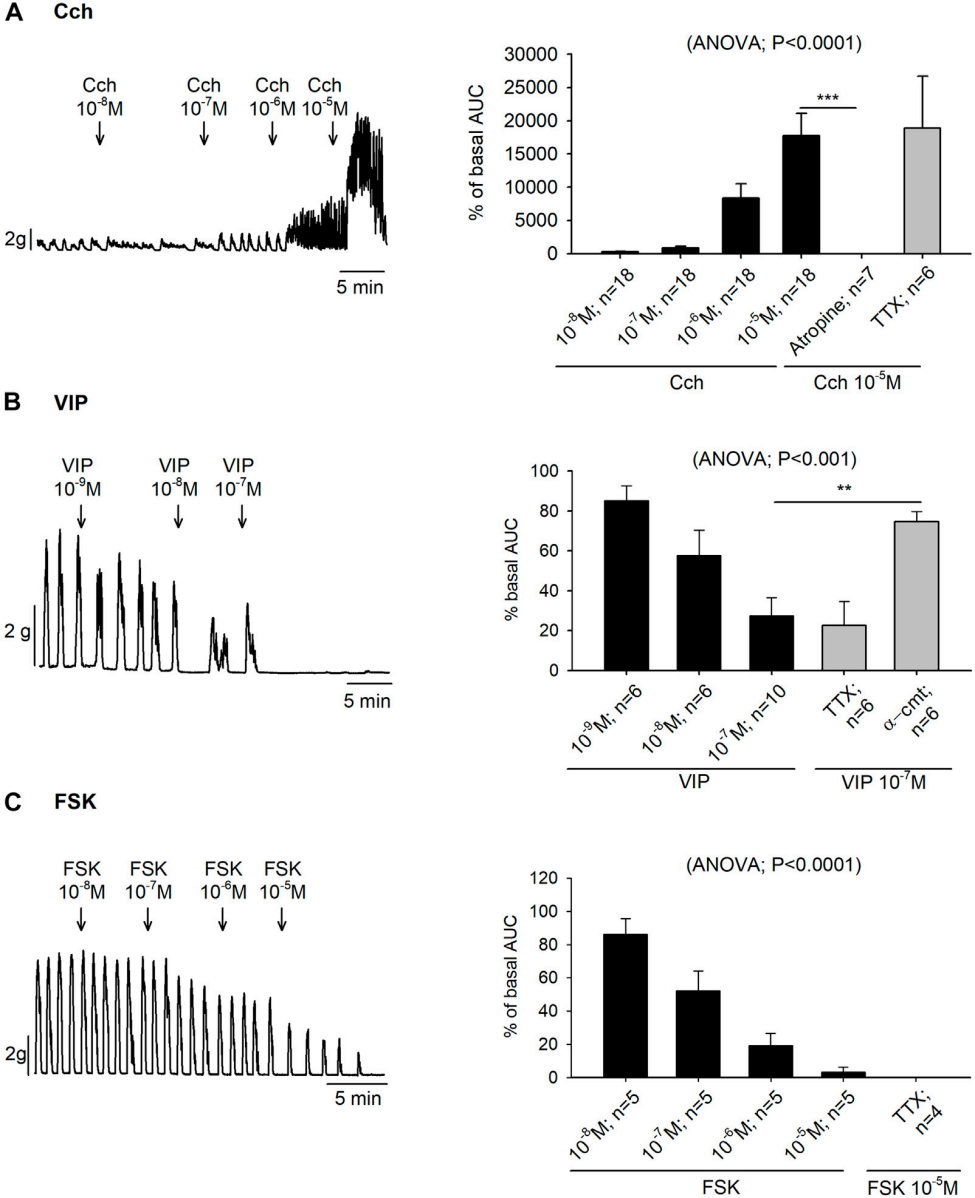
Effect of carbachol (Cch), vasoactive intestinal peptide (VIP) and forskolin (FSK) on colonic motility. Representative muscle bath recordings (left) of accumulative concentrations of Cch **(A)**, VIP **(B)** and FSK **(C)**, and histograms showing the effect of Cch **(A)**, VIP **(B)** and FSK **(C)** in control conditions (black) and after the incubation of atropine 10^−6^ M, TTX 10^−6^ M, or α-cmt 10U/mL before the addition of the highest concentration tested for each drug (grey) (right). Post Hoc test after ANOVA. **, *p* < 0.01; ***, *p* < 0.001. Data are expressed as mean ± SEM. n values represent strips from different animals.

Organ bath experiments showed that the increase of colonic contractions induced by Cch was also concentration dependent (10^–8^—10^–5^ M), with an EC_50_ of 1.24 µM. Prior incubation with TTX 10^–6^ M did not modify the Cch 10^–5^ M induced response but it was abolished with atropine 10^–6^ M (Figure 2A).

#### 3.1.2 Role of VIP and forskolin on colonic secretion and motility

VIP produced an increase of 17.15 ± 3.15 μA/cm^2^ ∆I_sc_ at 10^–7^ M. Tissue incubation with α-chymotrypsin (α-cmt) 10U/mL or with a Cl^−^ free Krebs solution, reduced VIP responses (Figure 1B). Forskolin (FSK) also increased the I_sc_ reaching 52.96 ± 11.61 μA/cm^2^ ∆I_sc_ at 10^–5^ M. The response was TTX insensitive, but it was strongly reduced when the preparation was incubated with a Cl^−^ free Krebs solution (Figure 1C).

In the colonic muscle layer, both VIP (10^–9^—10^–7^ M) and forskolin (10^–8^—10^–5^ M) produced a concentration-dependent decrease of the contractions with an EC_50_ of 0.022 µM and 0.12 µM, respectively. Both responses were TTX insensitive. Nevertheless, VIP response could be blocked with the pre-treatment of α-cmt 10U/mL (Figures 2B,C).

#### 3.1.3 Effect of nicotine on colonic secretion and motility

**FIGURE 3:**
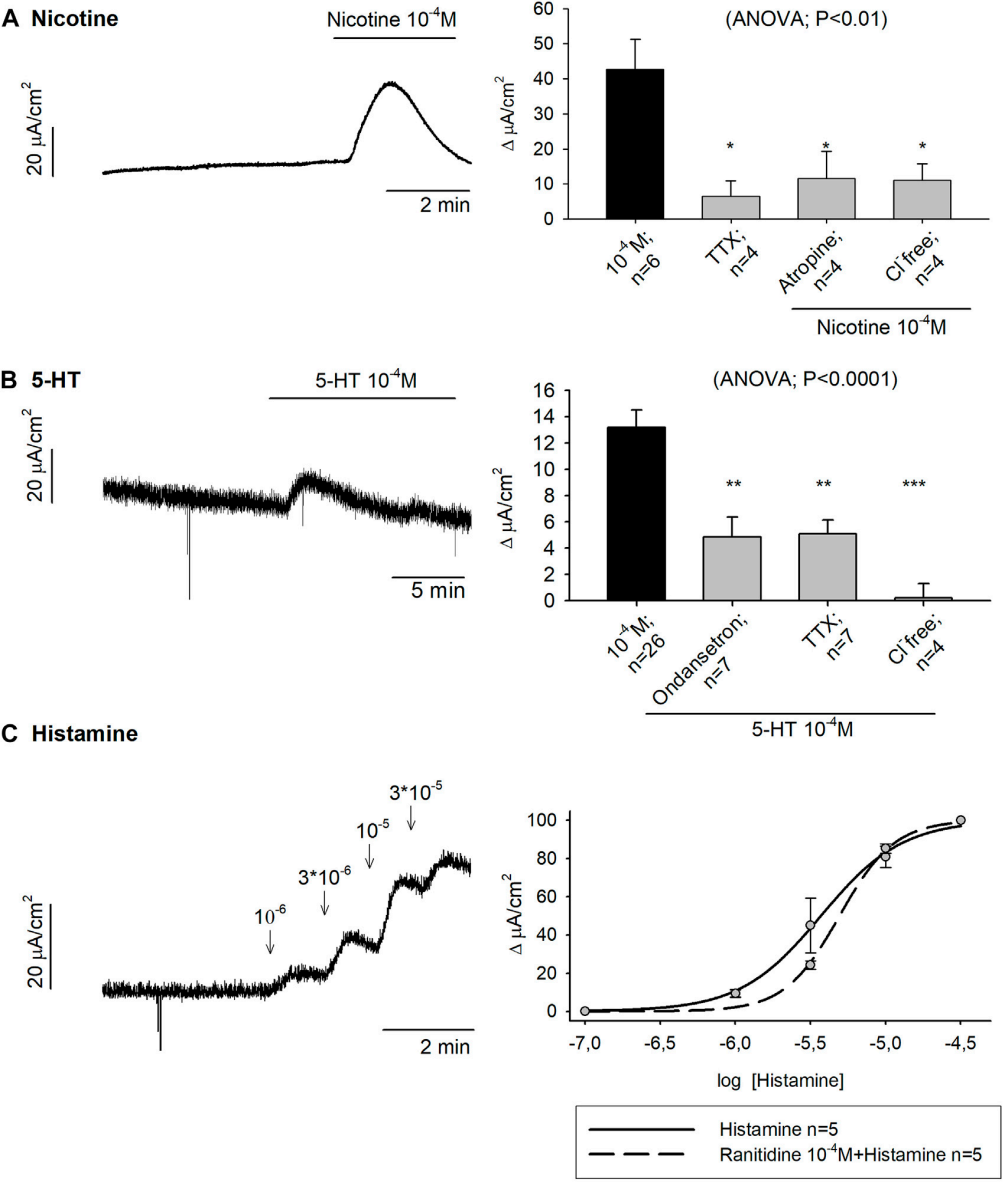
Effect of Nicotine, 5-HT and histamine on colonic secretion. Representative current recordings with Nicotine **(A)**, 5-HT **(B)** and Histamine **(C)** (left), and histograms showing the effect of Nicotine **(A)** and 5-HT **(B)** in control conditions (black) and after the incubation of TTX 10^−6^ M, atropine 10^−4^ M, Cl^−^ free solution or Ondansetron 10^−5^ M (grey) (right). Nonlinear regression curve showing the effect of Histamine **(C)** in control conditions (solid line) and after the incubation of Ranitidine 10^−4^ M (dashed line). Post Hoc test after ANOVA. *, *p* < 0.05; **, *p* < 0.01; ***p < 0.001 compared to the concentration tested for each drug under control conditions. Data are expressed as mean ± SEM. n values represent strips from different animals.

Nicotine 10^–4^ M produced 42.67 ± 8.66 μA/cm^2^ ∆I_sc_ in control conditions while a strong reduction of the response was observed when tissue was incubated with TTX 10^–6^ M, atropine 10^–4^ M and a Cl^−^ free Krebs solution (Figure 3A).

**FIGURE 4:**
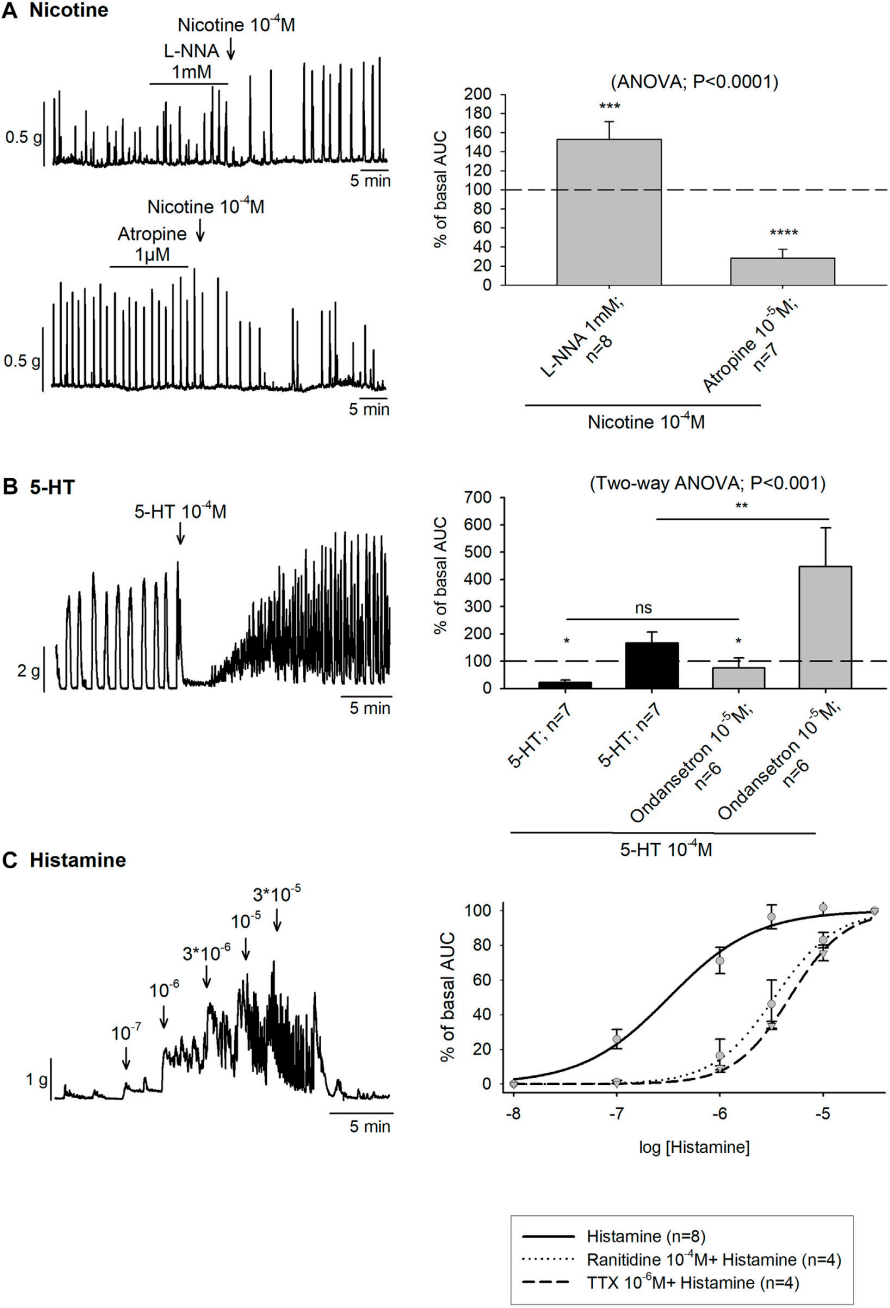
Effect of Nicotine, 5-HT and histamine on colonic motility. **(A)** Representative muscle bath recordings (left) and histograms (right) showing the contractile and inhibitory effect of Nicotine 10^−4^ M after the incubation of L-NNA 1mM or Atropine 10^−5^ M, respectively compared to the basal motility prior to drug addition. The (dotted line) is the basal motility (normalized to 100) prior to drug addition. **(B)** Representative recording of 5-HT 10^−4^ M and histogram showing the effect of 5-HT in the absence (black) and presence of ondansetron 10^−5^ M (grey) compared to the basal motility prior to drug addition (dotted line). The dotted line is the basal motility (normalized to 100) prior to drug addition. **(C)** Representative recording showing the effect of Histamine (left). Nonlinear regression curve showing the effect of Histamine in control conditions (solid line) and after the incubation of Ranitidine 10^−4^ M (dotted line) and TTX 10^−6^ M (dashed line) (right). n values represent strips from different animals.

Mechanical experiments were used to assess the presence of nicotinic receptors in enteric excitatory and inhibitory motor neurons using colonic strips incubated with L-NNA 1mM or atropine 10^–5^ M, respectively. In the presence of L-NNA 1 mM, an increase in colonic contractions was observed after nicotine 10^–4^ M addition. In contrast, prior incubation with atropine 10^–5^ M produced a substantial reduction of the colonic contractions (Figure 4A).

#### 3.1.4 Effect of serotonin on colonic secretion and motility

To study the role of 5-HT in the gut function, 5-HT 10^–4^ M was added to mucosal colonic preparations and 13.19 ± 1.30 μA/cm^2^ ∆I_sc_ was reached under control conditions. Subsequently, 5-HT 10^–4^ M was added in the presence of the 5-HT_3_ antagonist, ondansetron 10^–4^ M, TTX 10^–6^ M or a Cl^−^ free medium. In all cases, a reduction of the ionic secretion was observed (Figure 3B).

In the muscular layer 5-HT 10^–4^ M produced a biphasic response consisting of a first phase of relaxation, with a mean time duration of 3.85 ± 0.33 min followed by a second phase of contraction. However, when ondansetron 10^–4^ M was previously incubated, the first inhibition phase was less pronounced and significantly shorter, with a mean time duration of 2.26 ± 0.36 min (*t*-test, *p* < 0.01). Moreover, the AUC of the second phase increased reaching a mean AUC of 447.1% ± 141.6 % of basal AUC (Figure 4B). Thereafter, 5-HT was also studied in NNNP and TTX 10^–6^ M conditions. In both cases, the response consisted of only a phase of contraction (data not shown).

#### 3.1.5 Effect of histamine on colonic secretion and motility

Histamine was incubated in mucosal specimens in a concentration range of 10^–6^—3 × 10^−5^ M. The same concentration curve was conducted with prior incubation of the H_2_ antagonist, ranitidine 10^–4^ M. Histamine had an EC_50_ of 3.72 µM and when the H_2_ antagonist was incubated in advance, the EC_50_ was 4.93 µM (Figure 3C). Histamine 10^–5^ M was also incubated with a Cl^−^ free medium (n = 4) and, in this case, the secretory-induced response was almost abolished, with 6.12 ± 1.95 μA/cm^2^ ∆I_sc_ (*t*-test, *p* = 0.01) (data not shown).

In organ bath experiments, histamine increased the colonic contractions following a concentration-dependent pattern (10^–8^—3 × 10^−5^ M) with an EC_50_ of 0.32 µM. After ranitidine 10^–4^ M and TTX 10^–6^ M incubation, the EC_50_ was 3.37 µM and 4.74 µM, respectively although statistical differences were not observed in the Emax value (Figure 4C).

### 3.2 Characterization of the neurosecretory response

#### 3.2.1 Neural evoked mucosal chloride secretion

**FIGURE 5:**
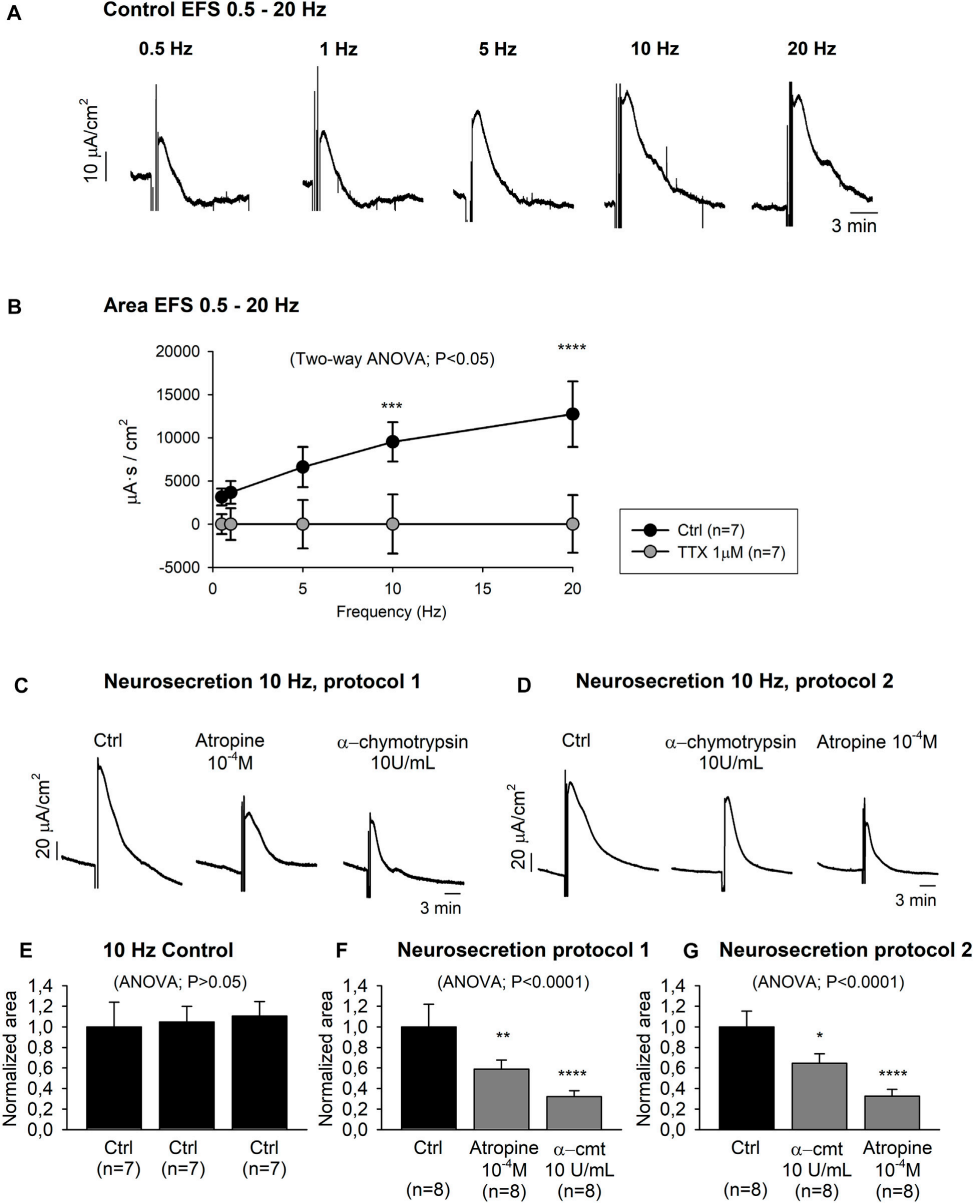
Neurally evoked mucosal chloride secretion. Representative current recordings **(A)** and graph **(B)** showing the effect of EFS (0.5–20 Hz) in control conditions and after TTX 10^–6^M incubation. Representative current recordings showing the effect of atropine 10^–4^ M and α-cmt 10U/mL and vice-versa (Protocol 1 and 2) on EFS (10Hz) induced responses **(C–D)**. Histograms showing three consecutive responses obtained in the same tissue in control conditions **(E)**. Histograms showing the effect of atropine 10^−4^ M and α-cmt 10U/mL (Protocol 1) **(F)** and vice-versa (protocol 2) **(G)**. Post Hoc test after ANOVA and Two-way ANOVA. *, *p* < 0.05; **, *p* < 0.01; ***, *p* < 0.001; ****, *p* < 0.0001. Data are expressed as mean ± SEM. n values represent strips from different animals.

Neurosecretory transmission was evaluated by EFS. Transmural EFS (0.5–20 Hz) evoked a frequency-dependent increase of the I_sc_ area from 3,139.90 ± 976.73 μA s/cm^2^ at 0.5 Hz to 12,738.06 ± 3,804.25 μA s/cm^2^ at 20 Hz (Figures 5A,B). I_sc_-EFS was significantly reduced when the same electrical stimulation protocol was carried out with the pre-treatment of TTX 10^–6^ M, indicating that the response was TTX-sensitive (Figure 5B).

Subsequently, 10 Hz EFS was used for further experiments to characterize the neural evoked response. It is important to notice that in our experimental conditions, 10Hz EFS elicited a TTX-sensitive submaximal response. Three consecutive 10 Hz EFS in the same preparation were reproducible, thus evoking similar I_sc_ responses (Figure 5E). Ion replacement experiments incubating colonic strips (n = 4) with a Cl^−^ free Krebs solution significantly reduced 10 Hz EFS response (Ctrl: 1.00 ± 0.24 normalized area, Cl^−^ free Krebs solution: 0.31 ± 0.05 normalized area) (data not shown). Thereafter, tissue was incubated with atropine 10^–4^ M and α-chymotrypsin (α-cmt) 10 U/mL (Neurosecretion protocol 1) or vice-versa (Neurosecretion protocol 2), and a cumulative reduction of the EFS-induced response was observed (Figures 5C,D,F,G). At the end of the experiment, TTX 10^–6^ M was incubated in both Neurosecretion protocols and almost a complete blockade of the EFS response was achieved (0.08 ± 0.01 normalized area and 0.13 ± 0.03 normalized area, respectively) (data not shown).

### 3.3 Effect of ETEC infection on secretion and motility

#### 3.3.1 Effect of ETEC infection on electrophysiological parameters and epithelial permeability

**FIGURE 6:**
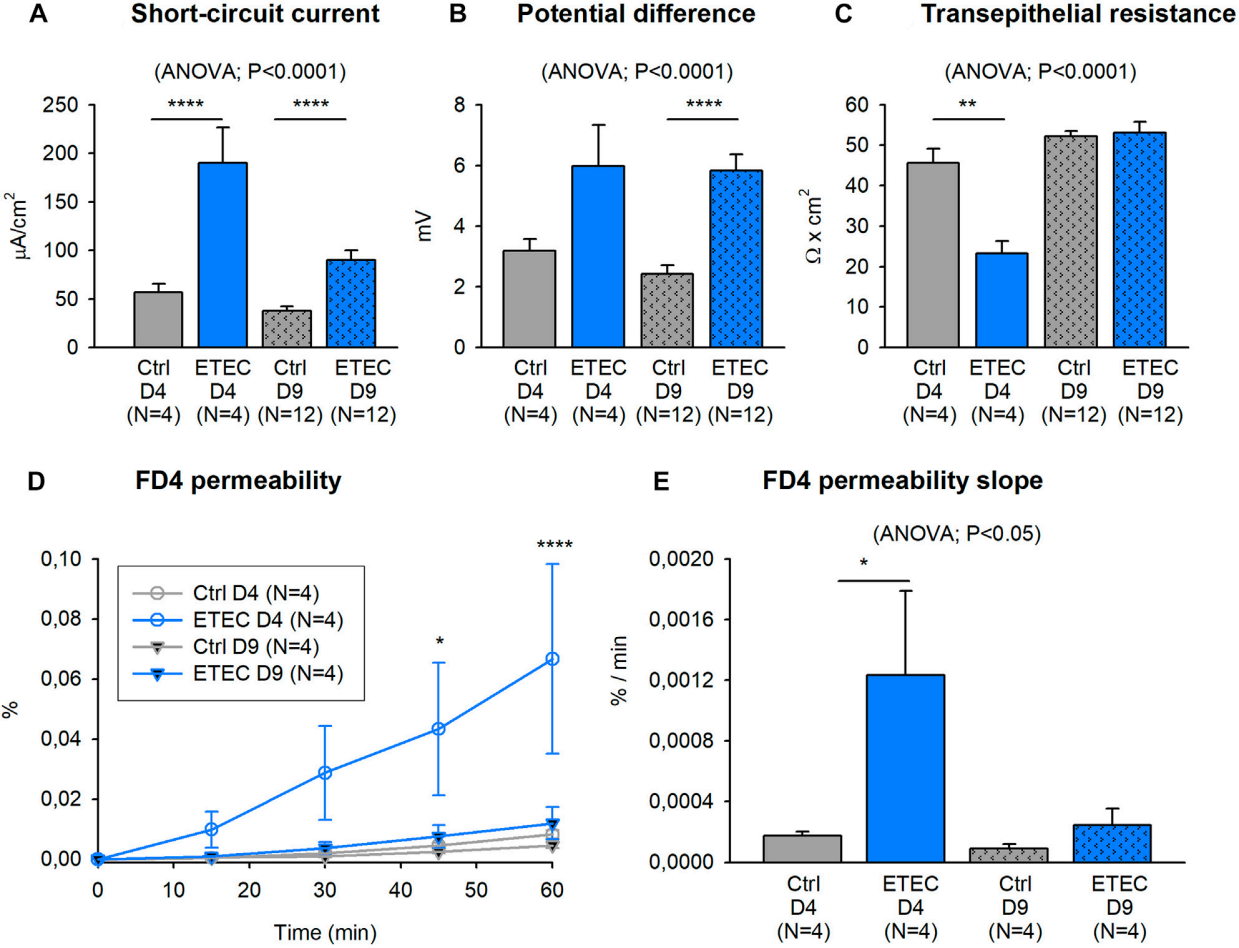
Effect of ETEC infection on electrophysiological parameters and epithelial barrier function. Histograms showing basal I_sc_, PD and TEER values 4- and 9-days of the ETEC challenge **(A–C)**. Graph showing the mucosal to basolateral passage of FITC-dextran (FD4) over 60 min concerning the amount added to the mucosal reservoir **(D)**. Histogram showing the FD4 flux to the basolateral compartment expressed as % of passage/min **(E)**. Post Hoc test after ANOVA and Two-way ANOVA. *, *p* < 0.05; ***, *p* < 0.001; ****, *p* < 0.0001. Data are expressed as mean ± SEM. N values represent different animals.

ETEC infection evoked an increase in the colonic active ion transport and consequently, higher basal I_sc_ and PD were observed in 4 and 9 days challenged animals compared to their respective controls (Figures 6A,B). TEER was measured as a colonic integrity indicator. In this case, a TEER reduction was observed at 4 but not at 9 days after the ETEC challenge (Figure 6C). In concordance, mucosal to basolateral passage of FD4 across colonic segments significantly increased in 4 but not in 9 days challenged animals. At day 4 post-challenge, ETEC produced an increase in the time course of the FD4 permeability compared with non-challenged animals, the kinetic of FD4 permeability reached statistical significance after 45 min of the incubation with FD4 (Figure 6D). Finally, the slope of the FD4 permeability also revealed a remarkable increase in the FD4 flux 4 days after the ETEC challenge (Figure 6E).

#### 3.3.2 Effect of ETEC infection on neural blockade on I_sc_

**FIGURE 7:**
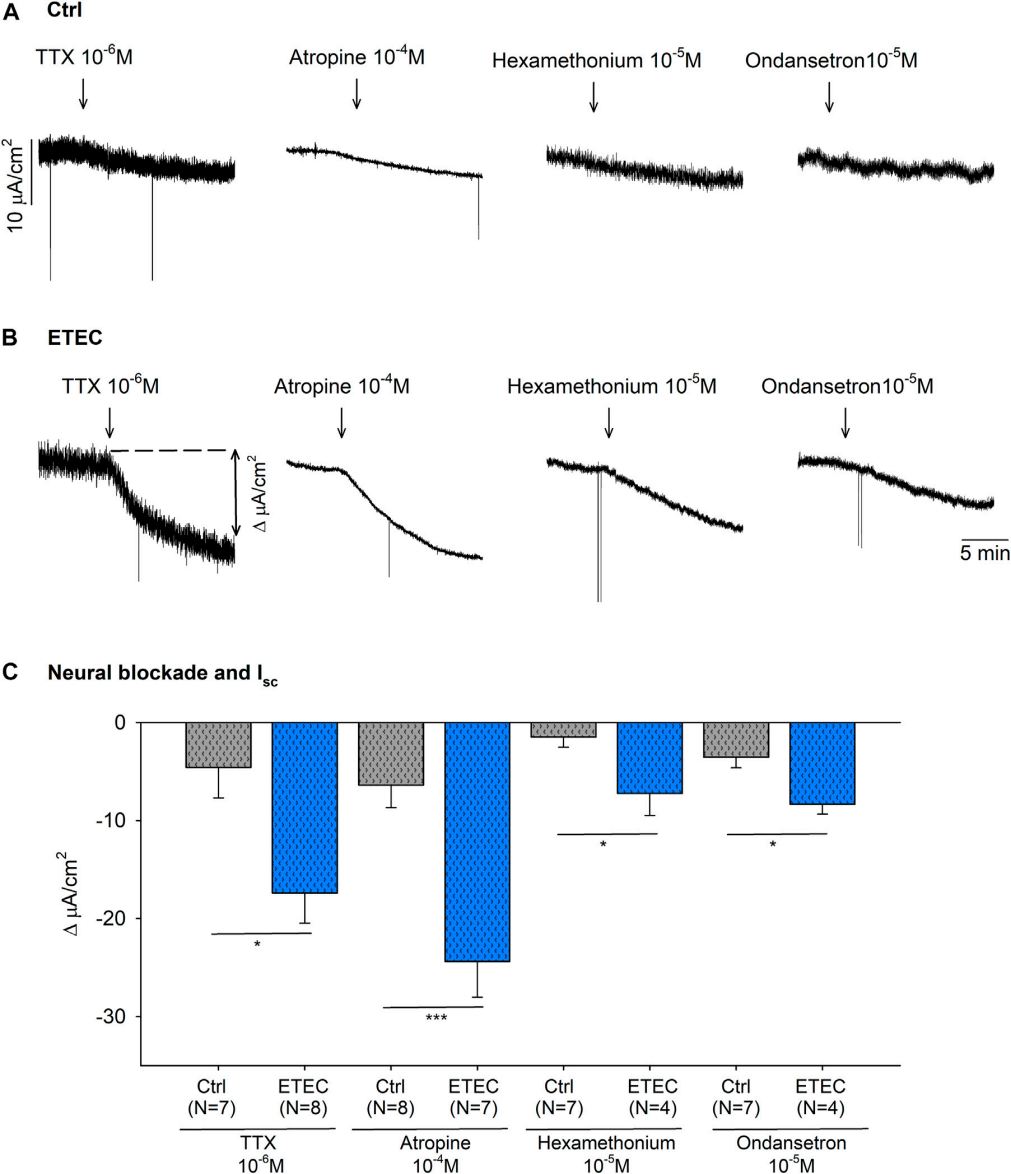
Effect of TTX 10^–6^ M, atropine 10^–4^ M, hexamethonium 10^−5^ M and ondansetron 10^−5^ M on the basal I_sc_. Representative current recordings **(A–B)** and histogram **(C)** showing the effect of TTX 10^–6^ M, atropine 10^–4^ M, hexamethonium 10^−5^ M and ondansetron 10^−5^ M in control conditions **(A–C)** and in 9-days post-ETEC challenged animals **(B–C)**. Histogram showing the effect of TTX 10^–6^ M, atropine 10^–4^ M, hexamethonium 10^−5^ M and ondansetron 10^−5^ M on the basal I_sc_ reduction at day 9 post-challenge. T-test. *, *p* < 0.05; ***, *p* < 0.001. Data are expressed as mean ± SEM. n values represent strips from different animals.

As seen in Figure 6, ETEC produced an increase in mucosal colonic electrolyte transport. Thus, colonic strips at day 9 post-challenge were incubated with a neural blocker, TTX 10^–6^ M, a muscarinic antagonist, atropine 10^–4^ M, a nicotinic antagonist, hexamethonium 10^−5^ M, and a 5-HT antagonist, ondansetron 10^−5^ M, to assess the role of neural activity in the basal ion transport. All of these produced a significant drop in the I_sc_ of ETEC-challenged strips (Figures 7B,C). In contrast, no significant reduction was observed in the I_sc_ of control animals after drug incubation (Figures 7A,C).

The incubation with both atropine 10^–4^ M and TTX 10^–6^ M produced a similar reduction of the I_sc_. Therefore, the I_sc_ value after the neural blockade with TTX 10^–6^ M and atropine 10^–4^ M were compared between ETEC and control animals. ETEC animals showed a higher I_sc_ after neural blockade compared to control animals (*p* < 0.01) (data not shown). This result suggests that the increased electrogenic ion transport reported in ETEC animals was not only due to a neurosecretory effect but also due to a direct epithelial effect of the ETEC.

#### 3.3.3 Effect of ETEC infection on muscular excitatory pathway

In order to eliminate neural inhibitory inputs, colonic tissue was incubated in Krebs solution using NNNP conditions. As expected, NNNP conditions increased spontaneous motility due to the presence of an inhibitory neural tone that was higher in both groups in the circular compared to the longitudinal muscle (Traserra et al., 2020). However, a similar increase of spontaneous motility between experimental groups was observed in both strip orientations (*p* > 0.05) (data not shown).

**FIGURE 8:**
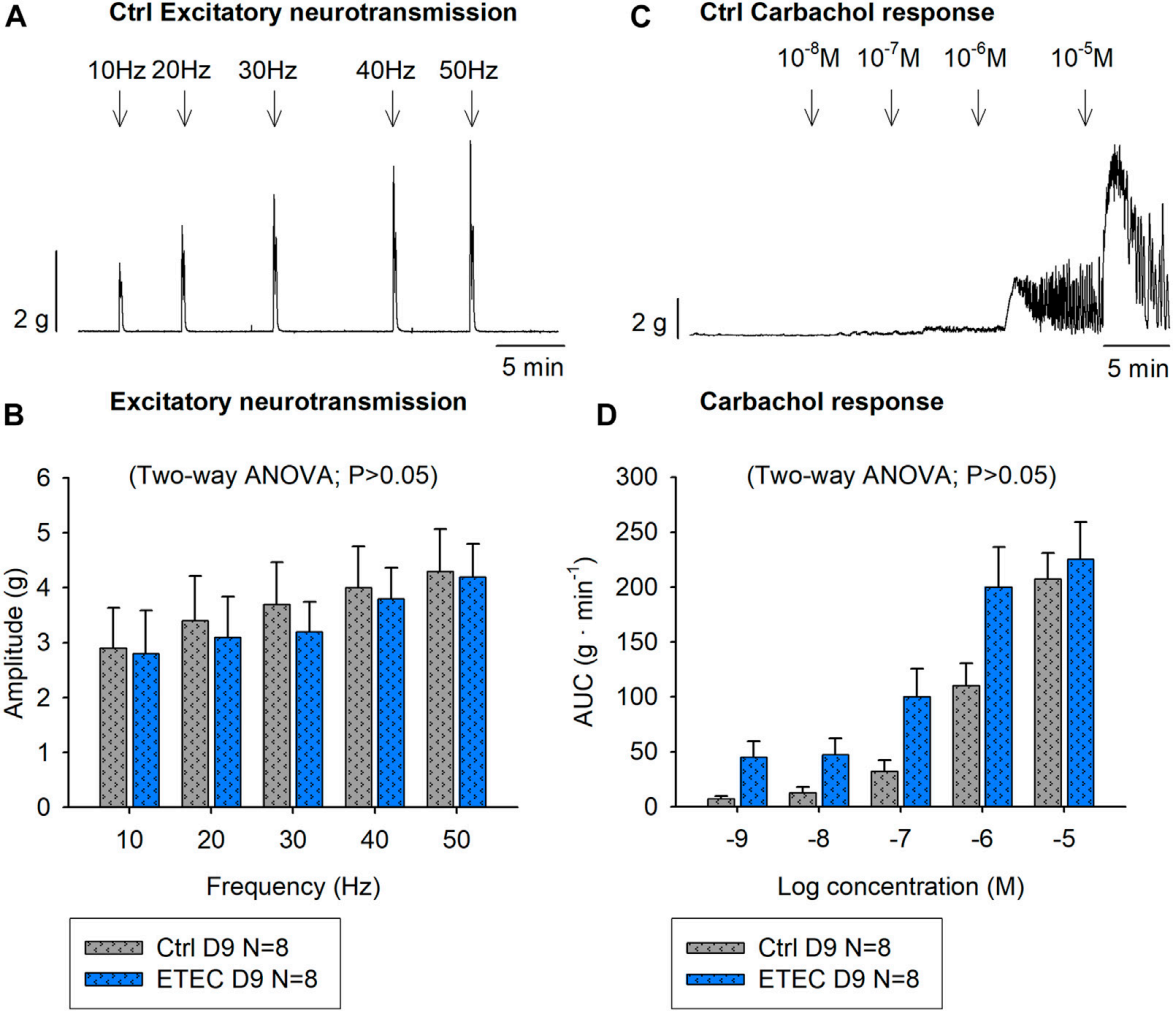
Effect of ETEC infection on the muscular excitatory pathway. Representative muscle bath recording from circularly oriented colonic strips from control animals **(A)** and histogram showing the effect of EFS (10–50 Hz) in 9-days post-ETEC challenged animals and in control animals **(B)**. Representative muscle bath recording from circularly oriented colonic strips from control animals **(C)** and histogram showing the effect of Cch (10^–8^—10^–6^ M) in control animals and in 9-days post-ETEC challenged animals **(D)**. Data are expressed as mean ± SEM. N values represent different animals.

NNNP conditions were used to selectively stimulate excitatory motor neurons that might be associated to contractile responses. Therefore, EFS was applied at increasing frequencies (10–50 Hz), which produced a frequency-dependent increase of the amplitude of neural colonic contractions. The response in both groups was TTX 1 μM and atropine 1 μM sensitive suggesting selective activation of excitatory motor neurons. No differences were observed between experimental groups at day-9 post-challenge in each colonic strip orientation (circular strips *p* = 0.8095, longitudinal strips *p* = 0.1132). In control animals, the highest frequency (50Hz) produced a mean amplitude of 4.3 ± 0.8 g and 4.5 ± 0.7 g in circular and longitudinal strips, respectively whereas ETEC-challenged animals reached a mean amplitude of 4.3 ± 0.6 g in circular strips (Figures 8A,B) and of 3.5 ± 0.5 g in longitudinal strips (data not shown). Moreover, atropine-sensitive concentration-response curves were obtained with the muscarinic agonist Cch (10^–8^—10^–5^ M), although no differences were observed between groups in either of the two colonic strips orientations (circularly oriented strips Two-way ANOVA, *p* = 0.0532 (Figures 8C,D); longitudinally oriented strips Two-way ANOVA, *p* = 0.1073) (data not shown).

#### 3.3.4 Effect of ETEC infection on colonic MCs

**FIGURE 9:**
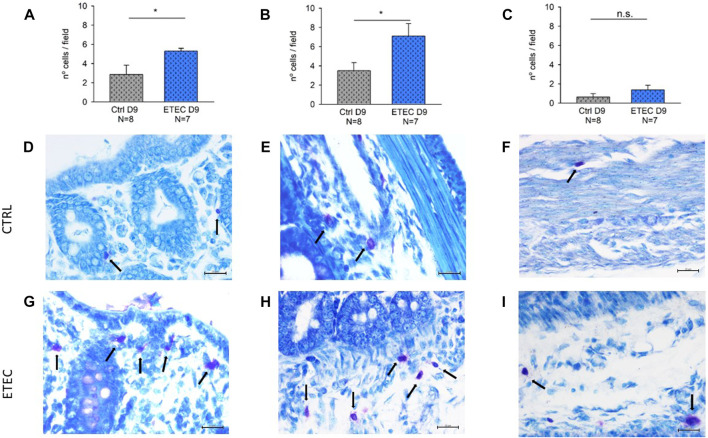
Effect of ETEC infection on the number of colonic MCs. Histograms showing the number of MCs per field in the mucosa **(A)**, the submucosa **(B)**, and the muscularis and serosa layers **(C)** in control animals and in 9-days post-ETEC challenged animals. Example of pictures from the mucosa, submucosa and muscularis and serosa layers, respectively from control animals **(D,E,F)** and from 9-days post ETEC challenged animals **(G,H,I)**. Counts and photos were obtained at 40x. Scale bar 20 μM. T-test. *, *p* < 0.05. Data are expressed as mean ± SEM. N values represent different animals.

MCs regulate epithelial function and integrity (Albert-Bayo et al., 2019). Accordingly, the presence of MCs was evaluated in three different colonic regions: the mucosa, the submucosa and the muscular and serosa layers. MCs were present in all the colonic layers, being predominantly in the submucosa and the mucosa in both experimental groups. However, differences were observed in the number of MCs per field between experimental groups on day 9 post-challenge. ETEC-infected animals presented a higher number of mucosal and submucosal MCs compared with their respective controls (Figures 9A,B,D,E,G,H) but non-significant differences were observed between groups in the muscular and serosa layers (Figures 9C,F,I).

## 4 Discussion

Intestinal absorption of water and electrolytes and formation and propulsion of feces toward the rectum involve epithelial and motor functions that are regulated by the ENS (Wood, 2004; Sharkey and Savidge, 2014). In this study, we examined in parallel the neural mechanisms associated with secretion and motility with the aim of assessing their possible involvement in ETEC-infected animals.

As expected, both Cch and VIP elicited TTX-resistant Cl^−^ secretion which was blocked respectively by the muscarinic antagonist atropine and α-cmt (Traynor et al., 1991), thus suggesting a direct effect on the enterocytes. However, these two molecules had opposite functions in the contractile experiments, since Cch elicited contractions and VIP relaxations (Kondo et al., 2011; Sanders, 2016). These results showed that ACh and VIP could be neurotransmitters released by secretory neurons, while ACh could be released by excitatory motor neurons and VIP by inhibitory motor neurons. These neurotransmitters could act directly at the effector level. Like the response elicited by VIP, forskolin evoked epithelial secretion and smooth muscle relaxation. Both effects are direct in the enterocytes and the smooth muscle layers, insensitive to TTX, and therefore, possibly due to an increase in cAMP in both effector cells thus mimicking the mechanism of action of enterotoxins (Muller and Baer, 1983; Schulzke et al., 2011; Krueger et al., 2013).

To assess the role of these neurotransmitters in neurosecretory and neuromuscular mechanisms, EFS was used to release neurotransmitters from intrinsic neurons in the intestinal wall. Mucosal EFS caused a transient increase of the I_sc_ that was strongly reduced by TTX and the combination of atropine and α-cmt, showing that ACh and VIP participate in neurosecretion. Therefore, this study elucidates, as previously reported in laboratory animals, that cholinergic and VIP-producing neurons are responsible for the EFS-evoked secretory responses in the pig colon (McCulloch et al., 1987; Kuwahara and Radowicz-cooke, 1988). The NOS inhibitor L-NNA was without effect (not shown) suggesting that in this model, neurosecretion is probably not due to NO, unlike in the human colon (Krueger et al., 2016). Neurochemical studies in the pig colon have revealed that ChAT neurons are the most abundant in the ISP, while the OSP is mainly characterized by the presence of nNOS and VIP in addition to ChAT neurons (Barbiers et al., 1993; Mazzoni et al., 2021). The differing chemical content between the neuronal populations of the ISP and OSP supports the hypothesis of a differentiated function between the two submucosal ganglionic plexuses, being ISP neurons most specifically involved in the control of the mucosa and OSP neurons in the control of both motility and epithelial processes (Timmermans et al., 1990). Regarding neuromuscular mechanisms, non-selective stimulation of intrinsic motor neurons causes virtually simultaneous activation of excitatory and inhibitory neurons. This produces responses that are difficult to interpret since there is a succession of so-called “on and off” responses (Mañé et al., 2015). To properly evaluate neuromuscular transmission, selective stimulation of excitatory and inhibitory neurons should be accomplished. Hundreds of papers have evaluated the identities of the inhibitory neurotransmitters using classical NANC conditions. We have been able to identify two major pathways, NO and purines acting on P2Y1 receptors as major inhibitory mediators in the porcine colon (Traserra et al., 2021). It is important to notice that despite the clear inhibitory effect of VIP, its role as an inhibitory neurotransmitter is difficult to demonstrate since IJP and corresponding relaxations are blocked with combinations of NOS inhibitors and P2Y1 blockers (Traserra et al., 2021). A functional role of VIP has been reported in the internal anal sphincter of the mouse (Keef et al., 2013). In our study, we propose to use NNNP pharmacological conditions to isolate the excitatory component of neurotransmission. Under these experimental conditions, the contractile response was blocked with TTX and atropine. These results should be interpreted as a rather selective activation of excitatory motor neurons that might be responsible for colonic propagated contractions.

Another way to stimulate intrinsic neurons is through the activation of nicotinic receptors. In this sense, nicotine binds to the receptor located on intramural neurons and produces an inward current that depolarizes them. This generates an action potential that releases the neurotransmitter at the synaptic level. In this study, we demonstrated the presence of nicotinic receptors in enteric neurons responsible for neurosecretion since the nicotinic response was reduced by TTX and atropine. Excitatory and inhibitory motor neurons responsible for contraction and relaxation also responded to nicotine since it produced contraction when the tissue was incubated with L-NNA and relaxation when it was incubated with atropine. Similar responses have been found in the human colon where nicotine produces activation of inhibitory neurons (Aulí et al., 2008). Together these results suggest the presence of nicotinic receptors in neurons responsible for motility and secretion.

Mechanical and chemical stimulation of the mucosa and wall distension triggers intrinsic reflexes due to the release of 5-HT that stimulates IPANs that contact with interneurons, secretory neurons and motor neurons (Camilleri et al., 2012). In this study, we demonstrated that the secretory response due to 5-HT was blocked by TTX and ondansetron suggesting the presence of 5-HT_3_ receptors in enteric neurons responsible for the neurosecretory circuit. 5-HT_3_ receptors are also involved in the control of motility since the inhibitory response was strongly reduced by ondansetron.

Control animals showed a baseline active ion transport similar to the results previously reported in post weaned pigs under physiological conditions (Boudry et al., 2004). However, electrophysiological parameters are dynamic and processes such as postnatal development, environmental stressors, or enterotoxins cause ongoing intestinal electrophysiological changes (Bach and Carey, 1994; Boudry et al., 2004; Moeser et al., 2007b). It is known that ETEC, once bounded, elaborates STa, STb and LT enterotoxins that stimulate different epithelial secretory signaling pathways that result in anion secretion and fluid loss (Dubreuil, 2012). In addition, the activation of the innate immune system in response to bacterial infection produces the release of MC-derived mediators, such as histamine, prostaglandins, 5-HT and proteases to fight the infection (Moeser and Blikslager, 2007; Wesolowski and Paumet, 2011). In this context, our study shows that animals’ exposure to ETEC diminished the integrity of the colonic barrier within the first days after the infectious challenge. This is consistent with the increase in permeability observed at 4 days post-infection and might be associated with a disruption in tight junctions (Nassour and Dubreuil, 2014). Moreover, an increase in I_sc_ was observed at day 4 post-infection that was partially reduced at day 9. It is important to notice that at day 9, epithelial secretion was still increased compared to controls, but permeability was normalized, which suggests a partial recovery of the mucosal barrier integrity with the persistence of increased Cl^−^ secretion. Also, MCs within the colon remained heightened until day 9 post-infection. These electrophysiological features were clinically manifested with diarrhea to flush the intestine of invading micro-organisms.

In our study, we have observed that the neurosecretory mechanism is mediated by the SMP because the preparations used had the serosal and muscular layers stripped. What is not known is whether the secretory reflex is integrated into the ISP or OSP. The fact that I_sc_ decreased by TTX demonstrated that the response is neurally mediated, as it has been previously described in other species such as in cats infected with enterotoxins (Cassuto et al., 1982), and rats infected with rotavirus (Lundgren et al., 2000). Our results also suggest that 5-HT, which might be released by EC cells, activates 5-HT-sensitive nerve endings, as reported in ETEC-infected rats (Mourad et al., 1995). The nicotinic antagonist, hexamethonium, also reduces the secretory response, which could explain the presence of synapse in the secretory pathway, as described in cats (Cassuto et al., 1982). According to our EFS data, the reflex pathway activates motorneurons that release mainly acetylcholine, and probably also VIP. Mourad and Nassad 2000 and Kordasti 2004, also postulated the release of VIP in the rat jejunum (Mourad and Nassar, 2000; Kordasti et al., 2004). In contrast to our results, (Cassuto et al., 1982), did not find an effect of atropine on response to cholera toxin (Cassuto et al., 1982). The movement of electrolytes over the epithelium is mainly mediated by luminal Cl^−^ secretion via a number of apical Cl^−^channels, mostly by the CFTR channel, but also by absorptive processes, especially of Na^+^ (Moeser and Blikslager, 2007). As we show in this study, this process is regulated by neurotransmitters and other non-neural mediators that act on receptors expressed on epithelial cells thus activating the second-messenger system (Xue et al., 2007). Therefore, the enterotoxins released by ETEC might have been involved in the activation of secretomotor reflexes that were blocked by TTX and atropine, indicating that cholinergic secretomotor neurons are the main neural component that regulates basal colonic secretion in ETEC challenged piglets (Schröder et al., 2010).

It has been also postulated that STb can increase intestinal peristalsis acting on the ENS to contract the smooth muscle (Moeser and Blikslager, 2007; Dubreuil, 2012). The increase in peristaltic activity may be due to two causes, an increase in motor activity due to neuromuscular contraction or due to a disinhibition mechanism, i.e., reduction of tonic inhibitory activity leading to contraction. Our experimental results showed that at day 9 post-challenge, when the epithelial barrier was restored, there were no differences between experimental groups in either of the two mechanisms since the excitatory neuromuscular activity in each of the two muscle layers did not differ and the presence of the inhibitory tone was similar between groups.

Luminal antigenic stimulation also produces MCs degranulation of inflammatory mediators, such as histamine (Thorbøll and Skadhauge, 1997; Ahrens et al., 2003; Albert-Bayo et al., 2019). In the GI tract, histamine is considered a regulator of GI functions through different histamine receptor subtypes. Besides, the response might be neurally mediated through the cross-talk between MCs and the ENS but also due to non-neural mediated pathways (Wood, 2006). In our experiments, histamine caused fluid secretion as previously reported in the pig colon (Ahrens et al., 2003). However, the response was not abolished in the presence of the H2 antagonist, ranitidine 10^−4^M. Prior studies have described the involvement of H1-receptors in the mucosal response to histamine in the guinea pig ileum (Cooke et al., 1984). In contrast, in the guinea pig distal colon and the pig proximal colon, the response to histamine has been reported to be H2-mediated (Cooke et al., 1984; Wang and Cooke, 1990; Ahrens et al., 2003; Wood, 2006). In the smooth muscle, histamine evoked a contractile response that was decreased by the H2 antagonist, ranitidine, and the neuronal conduction blocker, TTX. Nonetheless, prior studies of histamine’s contractile effect in the guinea pig colon, suggest it exerts this action via H1 receptors (Aguilar et al., 1986). In this context, we expect the contribution of other histamine receptor subtypes, rather than the H2 receptor, in the pig distal colon and the involvement of neural and non-neural pathways in the response.

In conclusion, in this study, we have characterized in parallel the neuronal pathways responsible for neurosecretion and contractility in the pig colon. We have shown that neurosecretion in the pig is due to ACh and VIP. 5-HT_3_ and nicotine receptors are probably involved in the neural pathways associated with motility and secretion. ETEC-infected animals showed transient alterations of the barrier and activation of neural mechanisms associated with increased secretion. Immunological mechanisms may also be involved in their activation, as mastocytosis was observed at the mucosal and submucosal level. In contrast to neurosecretory mechanisms, neuromuscular mechanisms associated with contraction and relaxation were not altered. This suggests that colonic motility should not be impaired in this type of infection and that diarrhea is primarily a secretory defense mechanism in which the ENS is actively involved. However, it cannot be ruled out that the increase in distension induced by diarrhea may end up producing a greater stimulus on motor phenomena which, as we can see in the present work, are totally conserved.

## Data Availability

The original contributions presented in the study are included in the article/supplementary material, further inquiries can be directed to the corresponding author.
